# Association of regional disparity of obstetrics and gynecologic services with children and infants mortality rates: A cross-sectional study

**Published:** 2017-03

**Authors:** Sogand Tourani, Mohammad Zarezadeh, Mehdi Raadabadi, Fatemeh Pourshariati

**Affiliations:** 1 *School of Health Management and Information Sciences, Iran University of Medical Sciences, Tehran, Iran.*; 2 *Students Scientific Research Center, Tehran University of Medical Sciences, Tehran, Iran.*; 3 *Social Determinants of Health Research Center, Shahid Sadoughi University of Medical Sciences, Yazd, Iran.*

**Keywords:** Obstetrics and gynecologic services, Children and infants mortality, Gini, Equity, Health Care

## Abstract

**Background:**

Equity in distribution of resources is considered as an important priority in health care systems. Equitable distribution of obstetrics and gynecology (Ob/Gyn) services in the country level is critical in maternal and neonatal health for qualitative promotion of maternal care in pregnancy, delivery, and post-delivery periods.

**Objective:**

The present study aimed at determining regional disparity of obstetrics and gynecology services and its association with children and infants mortality rates.

**Materials and Methods:**

This was a descriptive-analytical study conducted in 2015 to investigate distribution of Ob/Gyn services using three indicators of number of nursing and midwifery personnel, total Ob/Gyn specialists, and total delivery beds among 30 provinces of the country. Equity criteria in the present study included population, normal vaginal deliveries, cesarean sections, and total deliveries. Data were gathered using a researcher-made form and Stata 12 was used to calculate Gini coefficient. The association of Ob/Gyn services with children and infant mortality rates was investigated using SPSS package and linear regression test.

**Results:**

The lowest Gini coefficient was observed in distribution of nursing and midwifery personnel in delivery wards in terms of vaginal delivery (0.38 from 1) and the highest value was related to distribution of Ob/Gyn specialists in terms of vaginal delivery (0.73 from 1). Infant mortality was significantly associated with number of nursing and midwifery personnel in delivery wards, and total number of Ob/Gyn specialists.

**Conclusion:**

Considering new population policies in Iran and increased fertility rate, it is recommended to facilitate accessibility of the required services for the women, particularly those of reproductive age.

## Introduction

Health equity and elimination of health inequities have changed to one of the critical challenges in health care systems worldwide, particularly in developing countries (1). Regardless of different concepts, equity is considered as the core of health care system, hence the need for concentration on equitable distribution of services among different social groups (2). Equity in health has always been noticed as a permanent concern of researchers, planners, and policy makers in health care (3). Equality in distribution of health care sources and its effect on quality and quantity of services have long been challenged by the health policy makers. A large number of texts have defined equity beside access and equality (2, 4). 

According to Iran's 4^th^ and 5^th^ Development Plans and Iranian 2025 Vision Document, Ministry of Health is obliged to provide appropriate plans for equitable and need-based access to health care services. Also health care system is responsible and responsive to develop equity in different aspects and supply health care services according to need for care (5). General consensus suggests that inequality in access to health services is unfair and inequitable (6) and health care system is responsible to ensure equal access to health services according to need (7). An equitable service is one which is accessed according to need regardless of ability to pay (8). Socio-economic inequalities in using health care services result in increased burden of disease and exacerbates social inequalities in health leading to inappropriate socio-economic effects (9). 

Therefore, eliminating the gap in health inequalities in vulnerable populations has been recently taken into account (10). Investigations on utilization of health services help recognize access facilitating factors, causes of access barriers, and volume and quality of care provided (11). 2008 world health organization (WHO) report revealed that in 2006, 18329 children’s lives had been saved every day (12). In Iran, development of Primary Health Care along with increased admission of students in medical sciences have improved the country’s health status where human development index changed from 0.536 in 1990 to 0.702 in 2010 (13, 14). However, regarding all recent achievements and improved health indicators in Iran, differences of inequality pattern and promoting health indicators have not been taken into account as it seems that no specific plan is being performed in order to decrease inequality in children mortality rate (15). 

Also according to WHO report of 2009, children mortality rate in Iran was still 33 per 1000 live births which was about three times more than some neighboring countries such as Oman, Qatar, Bahrain, Kuwait, and Emirates (16). A brief look at the country health indicators in recent decade shows rapid growth of indicators, but on the other hand indicates inequalities in some indicators in different regions and provinces (15). In spite of such progress, neonatal mortality still persists. In most cases, it may be avoided by acknowledging maternal requirements within primary health care and by upgrading professional attendance with interventions to reduce the high number of deaths(17). Although child mortality coefficient has decreased from 120.7/1000 live births in 1970 to 19.9/1000 LB in 2010, neonatal mortality coefficient remains high, 13.6/1000 LB (68.0%). In fact, perinatal causes are potentially avoidable through access to qualified health services for the control of maternal diseases and complications in pregnancy and delivery (18).

Among all indicators, maternal health promotion is a critical base of health care services. From the very first years of foundation of health care system in Iran, mothers' health as the vulnerable group was specifically considered by health care policy makers. As the indicators of access to services improved, qualitative promotion of sufficient maternal care in pregnancy, delivery, and post-delivery seemed to be essential as a strategy to eliminate maternal mortality rate which was performed through taking an appropriate approach toward early detection and timely referral of mothers at risk (19). 

Women have a leading role in forming the culture and training as well as preserving and promoting family and society health. Therefore, optimal distribution of women health professionals such as obstetrics and gynecology (Ob/Gyn) specialists, midwives, and delivery beds in different regions of the country is of high importance, as women health guarantees family health and in a wider aspect society health (9). In this regard, the present study aimed at Regional Disparity distribution of obstetrics and gynecology services and its association with children and infants mortality rates in the country level.

## Materials and methods

This was a descriptive-analytical and cross sectional study conducted in 2015 based on the available data in a section of time in 2014. So All data is annually and 12 months were studied in 2014. The study population and sample composed of 30 provinces of Iran. Therefore, sampling was not performed and all the provinces were enrolled in the study.

Data gathering was conducted using a researcher-made form which consisted of the name of the province, population, number of normal vaginal deliveries, number of cesarean sections, total deliveries, number of nursing and midwifery personnel in delivery wards, total Ob/Gyn specialists, total delivery beds, infant mortality rate, under-one mortality rate, and under-five mortality rate. The data for all the provinces were collected from the Ministry of Health. 

**Figure 1:**
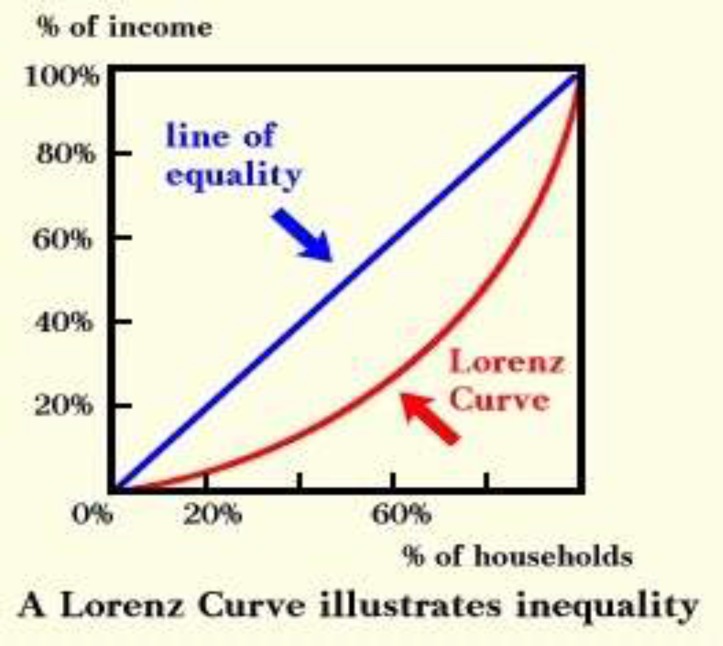
Lorenz curve

To investigate Ob/Gyn services inequalities, Gini index and Lorenz curve were used. Gini index is a criterion of inequality in distribution ranging between 0 and 1 which is the ratio of the area lying between the Lorenz curve and the line of equality over the total area under the line of equality (Figure 1) (20). Gini is an important index in assessment of equality being used in most of the similar studies which has a value of 0-1, 0 representing complete equality and 1 representing complete inequality. Gini index of <0.2 shows complete equality in distribution, 0.2-0.3 high equality in distribution, 0.3-0.4 inequality in distribution, 0.4-0.6 high inequality in distribution, >0.6 complete inequality. 

In this study, Gini coefficient was calculated using the following formula where y_i_ is the cumulated proportion of the variable (delivery bed, nursing and midwifery personnel, specialist) in the *i* province, x_i_ is the cumulated proportion of the population variable (i.e., vaginal delivery, C-section, total deliveries) in the provinces, n is total number of provinces.

$\mathrm{Gini coefficient}=\sum_{i=1}^{n}\left(y_{i=1}+y_{i})(x_{i+1}-x_{i}\right)$

**Ethical consideration**

Since the study was not conducted on animals and humans, so didn’t require getting a license from Ethics Committee.

**Statistical analysis**

In this study, in order to investigate distribution of Ob/Gyn services, three indicators of number of nursing and midwifery personnel in delivery wards, total number of Ob/Gyn specialists, and total delivery beds were assessed in terms of population, vaginal delivery, C-section, and total deliveries. 

Gini coefficient was calculated using these criteria by Stata 12. The association of Ob/Gyn services with children and infant mortality rates was investigated using Statistical Package for the Social Sciences, version 21.0, SPSS Inc, Chicago, Illinois, USA (SPSS) and linear regression test. 

## Results

Table I shows the values related to Ob/Gyn services and mortality rates of the provinces. The average of total deliveries was 40611.63, 51% of which were normal vaginal delivery and the rest were C-section. All Gini coefficients for Ob/Gyn services revealed inequitable distribution of this service in number of nursing and midwifery personnel in delivery wards, total number of Ob/Gyn specialists, and total delivery beds in terms of population, vaginal delivery, C-section, and total deliveries being more considerable in distribution of Ob/Gyn specialists (Table II). According to linear regression test, infant mortality was significantly associated with the variables of nursing and midwifery personnel in delivery wards, and total number of Ob/Gyn specialists, as infant mortality rate decreased by an increase in Ob/Gyn services (Table III).

**Table I:** Mean and SD of obstetrics and gynecology and mortality variables in the studied provinces (number & annually in 2014

**Variable (number & annually)**	**Min**	**Max**	**Mean**	**SD**
Population	545787	13422366	2349859.40	2486984.87
Normal vaginal delivery	3506	66583	20917.33	15755.86
C-section	3523	106275	19748.77	21067.40
Total deliveries	7029	159387	40611.63	34763.15
Nursing and midwifery personnel in delivery wards	20	1286	270.63	247.06
Total number of Ob/Gyn specialists	11	3615	314.37	820.80
Total delivery beds	62	2632	419.86	503.72
infant mortality	1	357	115.07	97.39
under-one mortality	10	750	186.67	161.79
under-five mortality	45	4526	1115.90	989.70

Ob/Gyn: obstetrics and gynecology

**Table II:** Gini coefficient for obstetrics and gynecology services based on population and delivery

	**Population**	**Vaginal delivery**	**C-section**	**Total deliveries**
Nursing and midwifery personnel in delivery ward	0.42	0.38	0.41	0.40
Total number of Ob/Gyn specialists	0.67	0.73	0.67	0.70
Total delivery beds	0.41	0.45	0.42	0.44

Ob/Gyn: obstetrics and gynecology

**Table III:** Association of obstetrics and gynecology services with mortality indicators

**Mortality indicators**	**Parameters**	**Nursing and midwifery personnel in delivery wards**	**Total number of Ob/Gyn specialists**	**Total delivery beds**
infant mortality	t	2.42	-2.91	-0.99
	Sig	0.023	0.007	0.331
under-one mortality	t	0.85	-0.40	-0.45
	Sig	0.402	0.692	0.653
under-five mortality	t	0.89	-0.32	-0.52
	Sig	0.380	0.750	0.601

Ob/Gyn: obstetrics and gynecology

**Figure 2:**
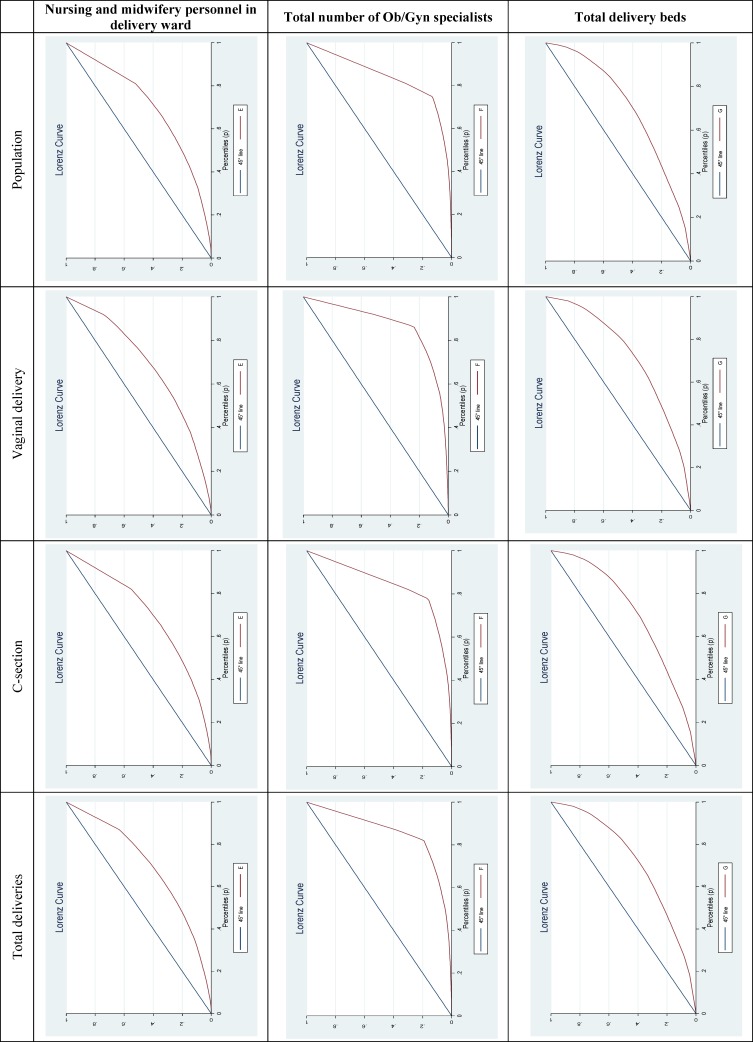
Lorenz curve of obstetrics and gynecology indicators

## Discussion

A review of Iran health care system status in late 1970s reveals extreme disparity in distribution of physicians throughout the country; as 69% of the specialists served in Tehran; 18% in Tabriz, Mashhad, Ahwaz, Shiraz, and Isfahan; and 13% in other cities (21). Appropriate and balanced distribution of resources particularly expert human resources as well as hospital beds in health care system can assist in promoting health indicators and consequently increasing equality and social equity. Mirsaeid and coworker. reported that despite increased rate of training expert human resources in health in recent years, many problems exist in an optimal distribution of these personnel in hospitals (22). In this regard, the current study was conducted to determine Regional Disparity of Ob/Gyn services and its association with children and infant mortality rates.

All Gini indices showed inequitable distribution of Ob/Gyn services including number of nursing and midwifery personnel in delivery wards, total number of Ob/Gyn specialists, and total delivery beds in terms of population, vaginal delivery, C-section, and total deliveries being more considerable in distribution of Ob/Gyn specialists. De Bruin *and colleague* investigated distribution of Coronary Care Unit beds in 24 university hospitals of the Netherlands from 2004-2006. Gini indices were found to be, respectively, 0.65 and 0.5 for the mentioned years indicating inequality in distribution of CCU beds (23). 

Horev *and colleague* studied trends in geographical disparities in allocation of resource in the US using Gini coefficient and reported a descending trend of equity in distribution of physicians to hospital beds during three decades. However, distribution of beds was found to be equitable (24). Olsen *and colleague *assessed inequality in distribution of health human resources in Tanzania and found inequitable and inefficient distribution of qualified staff (25). Using Gini coefficient, Matsumotoa and colleague found inappropriate distribution of physicians in Japan hospitals from 1996-2006 (26). According to linear regression test, infant mortality was significantly associated with the variables of nursing and midwifery personnel in delivery wards, and total number of Ob/Gyn specialists, as infant mortality rate decreased by an increase in Ob/Gyn services. Other studies showed that Ob/Gyn services in most Brazilian municipalities coped with the greatest number of high risk births and, therefore, the highest indexes of neonatal mortality (27, 28). 

The Disparity of obstetrics and gynecologic services for high risk obstetric attendance is very common in many countries. A multicenter analysis undertaken in eight Latin American countries (Argentina, Brazil, Cuba, Ecuador, Mexico, Nicaragua, Paraguay and Peru) reported that the public hospital was the most employed type of service (72.7%), which also featured the highest number of pre-term births and deaths in the neonatal period (29). Similar evidences could be seen in a neonatal unit in the public regional hospital of Valdivia, Chile (30). The opportune access to a whole set of obstetric and neonatal interventions, acknowledged for their efficaciousness, warrants the reduction of certain impairments and a higher survival rate of risk-prone newborns for a great percentage of the population. In fact, it is possible to attain a standard compatible with that of the best centers in the world (17).

The results of the present study and other similar works confirmed that regarding hospital beds and human resources as the basis of equity, there were significant inequities in the surveyed regions. Although solving the problems of equity in distribution of health resources and especially its measurement do not seem to be easy, this issue can highly affect policy making and resource allocation in health care systems (31). 

WHO has also emphasized the necessity of measuring equity in resource allocation. Considering access to health services as the fundamental right for all human being, inequality in geographical distribution of health resources negatively affects people access to these services (32). These problems seem more considerable in developing countries due to poor registration, collection, preservation, and analysis of data for planning in health care systems. Therefore, geographical equity and equity in distribution of health resources need to be emphasized as a public health index (33). One of the main reasons for lack of Ob/Gyn specialists in the province level is limited admission of students in Ob/Gyn specialty in the country. Some countries try to motivate health human resources financially to work in rural and deprived areas including offering scholarship, direct financial motivators, and offering loan to the physicians working in the mentioned areas (34-37).

Therefore, it is recommended that policy makers and other key decision makers in the Ministry of Health financially support the graduates of medical specialties and develop their educational and professional support. Since most of the women health services are provided by the Ob/Gyn specialists in pregnancy period, and policies of women health concentrate on pregnancy and post delivery services, thus these two indices are recognized as appropriate need indices for receiving health services related to Ob/Gyn specialists and midwifery staff (38).

## Conclusion

Regarding new population policies in Iran, it is predicted to have an increase in fertility rate in the coming years. This issue should be accompanied by proper planning for facilitating services for the women of reproductive age. Therefore, availability of the required services is recommended to be facilitated for the women particularly of reproductive age. Considering limited resources, related human resources should to be distributed based on need indices such as fertility rate, live births or fertility pattern of different regions. Also research is required to identify gaps within the different levels of health care to the mother and the neonate so that access inequalities could be eliminated.

## Conflict of interest

None declared. 
